# Co-Infections and Their Prognostic Impact on Melioidosis Mortality: A Systematic Review and Individual Patient Data Meta-Analysis

**DOI:** 10.3390/epidemiologia6020017

**Published:** 2025-04-01

**Authors:** Pakpoom Wongyikul, Wiyada Kwanhian Klangbud, Moragot Chatatikun, Phichayut Phinyo

**Affiliations:** 1 Center for Clinical Epidemiology and Clinical Statistics, Faculty of Medicine, Chiang Mai University, Chiang Mai 50200, Thailand; aumkidify@gmail.com (P.W.); phichayutphinyo@gmail.com (P.P.); 2 Department of Biomedical Informatics and Clinical Epidemiology (BioCE), Faculty of Medicine, Chiang Mai University, Chiang Mai 50200, Thailand; 3 Medical Technology Program, Faculty of Science, Nakhon Phanom University, Nakhon Phanom 48000, Thailand; 4 School of Allied Health Sciences, Walailak University, Nakhon Si Thammarat 80160, Thailand; morragot.ch@wu.ac.th

**Keywords:** melioidosis, coinfection, prognostic factor, mortality, systematic review, meta-analysis

## Abstract

**Objectives**: This study aimed to evaluate the prognostic impact of coinfections and other clinical factors on mortality in melioidosis patients, providing a comprehensive analysis through systematic review and meta-analysis. **Methods:** A systematic search was conducted in PubMed, Embase, Scopus, and other sources for studies published from their inception to August 2023. Studies reporting mortality outcomes in melioidosis patients with and without coinfections were included. Mixed-effects logistic regression models were used to estimate the causal association of each prognostic factor on the outcome. Directed acyclic graphs (DAGs) were used to guide confounding adjustment, and missing data were handled using multiple imputations. **Results:** A total of 346 studies involving 509 patients were analyzed. Coinfections were observed in 10.8% of patients with tuberculosis and *Leptospira* spp. being the most common. Disseminated disease significantly increased the odds of death (OR 4.93, 95% CI: 2.14–11.37, *p* < 0.001). Coinfections were associated with a higher mortality rate, but the association was not statistically significant (OR 2.70, 95% CI: 0.53–13.90, *p* = 0.172). Sensitivity analyses confirmed the robustness of the findings. Other factors, including diabetes mellitus and agricultural occupation, were evaluated for their associations with mortality. **Conclusions:** Disseminated melioidosis remains a significant factor influencing prognosis. Although less common, coinfections may contribute to worsen patient outcomes, emphasizing the importance of immediate and accurate diagnosis and comprehensive management.

## 1. Introduction

Melioidosis, caused by the gram-negative bacterium *Burkholderia pseudomallei*, represents a major global health concern, particularly in tropical regions such as Southeast Asia, northern Australia, and South America [1]. This disease is characterized by a wide range of clinical manifestations, from localized skin infections to severe, life-threatening conditions such as septic shock and multiorgan failure. The clinical spectrum of melioidosis reflects the pathogen’s ability to affect multiple organ systems and its capacity to cause both acute and chronic illness [2].

The disease is further complicated by its association with various coinfections and underlying health conditions, which can significantly impact disease progression and patient outcomes [3]. Coinfections with Mycobacterium tuberculosis [3,4], *Leptospira* spp. [5,6], and human immunodeficiency virus (HIV) [7] have been reported in melioidosis patients and are known to influence the severity of the illness. For example, tuberculosis coinfection has been linked with increased mortality, possibly due to its interaction with *B. pseudomallei* in immunocompromised individuals [8,9]. Similarly, coinfections with *Leptospira* have shown a high fatality rate [10], highlighting the need for a better understanding of how these interactions affect patient outcomes.

Underlying conditions, particularly diabetes mellitus (DM), are also significant contributors to the severity of melioidosis [11]. Diabetes is a known risk factor for both susceptibility to and poor outcomes from melioidosis [1,9,11]. Chronic diseases and socioeconomic factors may also affect disease severity and mortality [11]. These factors potentially alter the immune response, disease progression, and treatment efficacy, making it crucial to explore their combined effects [1,9,11].

Although there are known associations between coinfections and underlying conditions with melioidosis severity [3,4,5,6,7,8], a comprehensive evaluation of their prognostic impact on mortality is lacking. Previous studies have focused primarily on individual factors without systematically analyzing their collective influence. This gap in research necessitates a thorough investigation to elucidate how coinfections and other prognostic factors interact and contribute to the overall mortality risk in melioidosis patients. This systematic review and meta-analysis aim to address this knowledge gap by synthesizing data from various studies to assess the impact of coinfections and other prognostic factors on melioidosis mortality. By aggregating and analyzing data on patient characteristics, coinfection status, and disease outcomes, we aimed to better understand the factors influencing mortality in patients with melioidosis, regardless of other variables. Our findings will offer valuable insights into how coinfections and underlying conditions affect disease severity, potentially guiding future research and clinical practice. Understanding these relationships is critical for improving diagnosis, treatment strategies, and preventive measures, ultimately enhancing patient outcomes and managing the burden of melioidosis in affected regions.

## 2. Materials and Methods

### 2.1. Protocol and Registration

This systematic review was undertaken and reported following current methodological standards and the Preferred Reporting Items for Systematic Reviews and Meta-Analyses (PRISMA) guidelines [12]. The study protocol was registered with the International Platform of Registered Systematic Review and Meta-analysis Protocols (INPLASY), and the registration number is INPLASY2024120013.

### 2.2. Search Strategy

A comprehensive, systematic literature search was conducted in PubMed, Embase, Scopus, and alternative sources (Google Scholar) for studies published from database inception to August 2023. Additionally, the reference lists of relevant studies were screened for further eligible studies. Two researchers independently screened, reviewed, and extracted data, excluding duplicate articles. Our search terms for the coinfection group included “*Burkholderia pseudomallei*”, “*B. pseudomallei*” “Melioidosis”, “coinfection”, “cooccurrence”, “superinfection”, “clinical outcome”, and “clinical manifestation”, along with their specific controlled vocabularies for each database. Moreover, the single-infection group did not include the terms “coinfection” and “cooccurrence”.

### 2.3. Inclusion and Exclusion Criteria

Only studies published in English were included. The review focused on case reports, case series, cohort retrospective studies, and prospective observational studies. Case reports and case series were included if they detailed coinfections and their impact on mortality in melioidosis patients. The cohort retrospective and prospective observational studies were included if they examined how coinfections and other prognostic factors influenced mortality on the basis of historical patient data. Studies were excluded if they did not provide substantial data, such as conference abstracts, editorials, or opinion pieces. Research lacking specific data on mortality, coinfections, or that did not directly address melioidosis was also excluded. Additionally, studies with incomplete data that could not be meaningfully analyzed were excluded to ensure the accuracy and reliability of the review.

### 2.4. Data Extraction Process and Outcome

Details related to the following data were extracted from each study: first author’s name, study type, publication year, country of origin, number of patients, patient’s age, sex, occupation, underlying condition, type of infection, and dissemination of disease. Two reviewers (WKK and PW) applied the eligibility criteria and selected studies for inclusion in the systematic review. These reviewers independently screened records for inclusion. Disagreements between individual judgments were resolved by a third reviewer (PP). The primary outcome was death from any cause (mortality).

### 2.5. Conceptual Causal Diagram

We aimed to assess the causal associations for each prespecified factor of interest, including publication year, socioeconomic country status, age group, sex, agricultural occupation, diabetes mellitus, other underlying diseases (if applicable), type of infection, and disease dissemination with mortality in patients with melioidosis. Therefore, confounding factors had to be identified and fully controlled. We constructed a causal diagram based on prior evidence, available data, and expert opinions to display the conceptual causal relationships among these factors. Each factor of interest had its own set of confounders for adjustment and its model. For example, when assessing the causal association between agricultural occupation (determinant) and mortality (outcome), adjustments were made for age and socioeconomic country status (as confounders). In contrast, a different set of confounders was used to analyze sex (the determinant). The causal diagram was generated via the directed acyclic graph (DAG) method via the browser-based tool DAGitty [13].

### 2.6. Statistical Analysis

Categorical data on study-level and patient-level characteristics are reported frequencies and percentages. The death rate for each prognostic factor was expressed as a percentage of the total number of patients in the same prognostic category. As an individual patient data (IPD) meta-analysis including data from case reports and case series, we used a one-stage multilevel mixed-effects logistic regression to estimate the odds ratio for each causal relationship model.

We first assessed the validity of complete case analysis (CCA) to handle missing data to determine whether it was likely to produce biased results via James R. Carpenter’s framework [14,15]. It would be applied if the evaluation indicated that the CCA was unlikely to introduce bias. In this case, we performed a sensitivity analysis via multiple imputation (MI) with chained equation (MICE) [16]. This sensitivity analysis aimed to compare the model efficiency between the CCA and MI methods, maximizing the use of partially observed data. If CCA was deemed invalid, the results from the MI were applied.

We conducted a post hoc sensitivity analysis to evaluate the robustness of our results when relying solely on evidential support for the causal diagram and model. The Supplementary Materials provides details on the definition of evidence level and the rationale for conducting this analysis.

## 3. Results

### 3.1. Results of the Search

**Figure 1:**
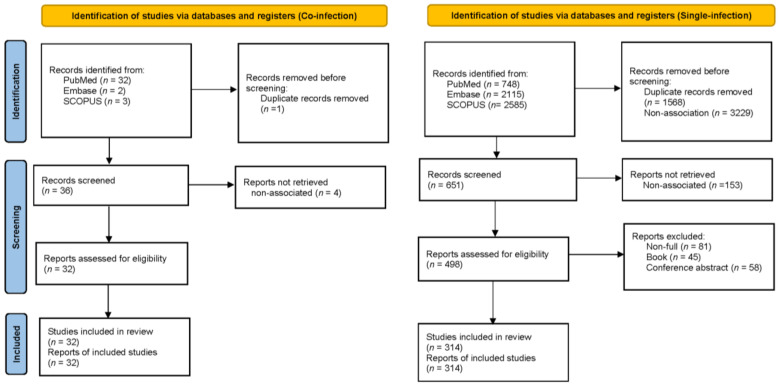
PRISMA flow.

Melioidosis coinfection studies refer to studies investigating the impact of additional infections on melioidosis mortality. The search process identified a total of 37 records from three databases: PubMed (*n* = 32), Embase (*n* = 2), and SCOPUS (*n* = 3). After one duplicate record was removed, 36 studies were screened, and 4 studies were excluded because of nonassociation. This screening led to 32 reports being assessed for eligibility. All 32 studies were deemed eligible and were included in the systematic review and meta-analysis. In contrast, single-infection studies focus on melioidosis without any additional coinfections, serving as a baseline for comparison. A total of 5448 records were identified across PubMed (*n* = 748), Embase (*n* = 2115), and SCOPUS (*n* = 2585). After removing 1568 duplicate records and 3229 unrelated to the topic, 651 records were eligible for screening (the references are in Supplementary File S1). Next, 81 nonfull reports, 45 books, and 58 conference abstracts were excluded, leaving 498 reports for eligibility assessment. Among these, 314 studies were included in the systematic review and meta-analysis, as shown in the PRISMA flow (Figure 1).

### 3.2. Study Characteristics

This systematic review and meta-analysis included 346 studies examining coinfections and prognostic factors in melioidosis. Among these studies, 58.4% were published between 2016 and 2023, with the remaining 41.7% published before 2016. The extracted data of all studies are shown in Supplementary File S2. The studies were conducted across high-, upper-middle-, and lower-middle-income countries as shown in Table 1.

Data from 32 studies covering 55 patients [3,4,5,6,7,10,17,18,19,20,21,22,23,24,25,26,27,28,29,30,31,32,33,34,35,36,37,38,39,40,41,42] were analyzed for coinfection patients. Notably, most coinfection studies were published before 2016, and 35.5% came from upper-middle-income countries. Coinfection patients tended to be younger, with 47.3% in the 19–49 years age group, and a significant proportion (81.8%) were male. Additionally, 61.8% of coinfection patients presented with disseminated disease, which is a critical factor associated with increased mortality.

For single-infected patients, 454 individuals were included in 314 studies, with 60.4% of the studies published after 2016. A larger share of single-infection studies (35.9%) came from high-income countries. Single-infected patients were older, with 45.4% aged 50 years or older. Like in the coinfection group, most patients were male (68.7%), and over half (55.5%) had disseminated disease.

### 3.3. Patient Characteristics

The patient population included in this analysis comprised a total of 509 individuals. The mean age was 45.4 ± 18.1 years, with 11.9% under 18 years, 43% between 19 and 49 years, and 45.1% aged 50 years or older. Most patients were male, accounting for 70.1%, whereas 29.9% were female. Notably, 18.9% of patients were engaged in agricultural occupations. Diabetes mellitus (DM) was prevalent in 51.5% of the patients, and 39.3% had other underlying health conditions (Table 1).

In terms of infection type, 89.2% of the patients had single-infections caused by *Burkholderia pseudomallei*, whereas 10.8% experienced coinfections. Tuberculosis (TB) was the most common coinfection (18 patients), followed by *Leptospira* species and HIV. Additionally, 56.2% of patients had disseminated infections, whereas 43.2% presented with non-disseminated disease. *Leptospira* had the highest proportion of fatalities (Table 2).

### 3.4. Missing Data

Nine prognostic factors were assessed for their association with death. The year of publication and sex were reported. However, information on agricultural occupation status was reported for only 39.3% (200 individuals) of the total individual patient data. Minimal missing data were observed for the other prognostic factors, ranging from 0.1% to 1.8% (Table 1). We analyzed the data to assess the validity of the CCA. The missingness of agricultural occupation data did not depend on the outcome (death); therefore, the CCA is valid in this study.

### 3.5. Causal Associations Between Prognostic Factors and Death

#### 3.5.1. Complete Case Analysis

Supplementary Table S1 presents the causal model, adjusted set, and assessment of the validity of CCA for each factor. For the study-level prognostic factors, a publication year after 2015 demonstrated a reduction in the death rate. Compared with high-income countries, upper-middle-income countries were associated with a greater risk of death, whereas the lower-middle-income countries presented a similar risk (Table 3). However, these findings were not statistically significant.

In terms of patient-level prognostic factors, older age, male sex, and agricultural occupation were associated with a trend toward a reduced risk of death. The findings were not statistically significant. Four prognostic factors, namely, DM, the presence of other underlying factors, coinfection type, and dissemination of disease, were associated with a greater risk of death. However, only the dissemination of disease was statistically significant (OR 4.93, 95% CI: 2.14–11.37, *p*-value < 0.001) (Table 3). The details of each causal diagram are provided in Supplementary Figures S1–S9.

#### 3.5.2. Multiple Imputation Sensitivity Analysis

**Figure 2:**
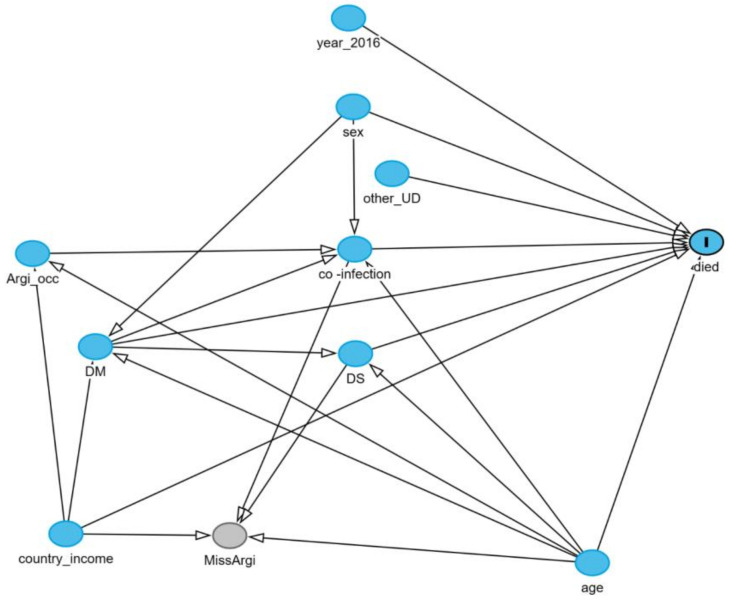
Conceptual causal diagram. A diagram showing a causal relationship between each two variables indicated by black arrow MissArgi, a binary variable that indicates whether agricultural occupation is observed or missing. Abbreviation: Argi_occ, agricutural occupation; country_income, socio-economics country status;DS, disseminated, DM, diabetes mellitus, other_UD, other underlying disease; year_2016, the publication year after 2016.

Seven prognostic models were assessed for sensitivity analysis via multiple imputations (MI) in Table 3. Two of seven models, including socioeconomic country status and age groups, exhibited an association with death similar to that of the complete case analysis (CCA). Three models, including DM, other underlying and types of infection, were associated with death at higher risk. While agricultural occupation and the dissemination of disease exhibited an association with death at lower risk than CCA. The conceptual causal diagram of the study is shown in Figure 2. Overall, the sensitivity analysis based on multiple imputations revealed slight differences in the magnitude of association and confidence interval width for these prognostic factors. The conclusions of the causal associations remained the same (Table 3).

#### 3.5.3. Post Hoc Sensitivity Analysis for Only Evidential Support Causal Diagram

We performed a sensitivity analysis to eliminate certain pathways between variables on the basis solely of expert opinions and available data (Supplementary Table S2). Three causal relationships between sex and coinfection type, agricultural occupation and coinfection type, age group, and agricultural occupation were identified (Supplementary Figure S10). However, only Model 5 had different adjustment sets (adjusted only for socioeconomic country status), and the magnitude of association was similar to that of the model based on the conceptual causal diagram (Supplementary Table S3).

## 4. Discussion

The findings of this systematic review and meta-analysis contribute significantly to understanding the multifaceted factors influencing mortality in patients with melioidosis. The high mortality associated with disseminated disease reinforces its critical role as a prognostic marker. Dissemination likely indicates severe systemic immune dysfunction, allowing *Burkholderia pseudomallei* to invade multiple organ systems, complicating management and increasing the likelihood of poor outcomes [42]. This finding aligns with existing evidence suggesting the urgent need for early detection and aggressive treatment of disseminated infections to mitigate mortality risk [43].

Coinfections, while less frequently reported, exhibited a notable trend toward higher mortality, emphasizing their potential to exacerbate the clinical course of melioidosis. Tuberculosis [3,4,29,30,31,32,33,34,35,41,42] and *Leptospira* spp. [5,6,10,26,27,37] are the predominant copathogens, which is consistent with their prevalence in endemic regions. These pathogens may amplify inflammatory responses, leading to cytokine storms [44,45,46,47,48] and immunological exhaustion [49,50,51], which compound the severity and fatality rate of melioidosis [52]. The particularly high fatality rate associated with *Leptospira* spp. coinfection (75%) suggests synergistic pathogenic effects that demand further investigation. However, the lack of statistical significance in coinfection-related mortality highlights the need for more extensive, well-powered studies to better elucidate these relationships [53].

Coinfection with Mycobacterium tuberculosis is among the most frequently reported infections in melioidosis patients [3,4,29,30,31,32,33,34,35,41,42]. The immune-modulating effects of tuberculosis are well-documented and are often characterized by chronic granulomatous inflammation that suppresses effective pathogen clearance [54]. This may exacerbate the pathogenicity of *B. pseudomallei*, which thrives in a compromised immune microenvironment [55]. The synergistic effect of these pathogens could lead to overlapping clinical manifestations, such as pulmonary or systemic involvement [3,4], complicating diagnosis and delaying appropriate treatment. Additionally, tuberculosis-induced immunosuppression [51,55], particularly in the context of latent infections, may be activated or worsened by melioidosis. For example, shared reliance on IFN-gamma-mediated immunity may result in competitive immune resource depletion [8,56]. Furthermore, because both pathogens can be latent, especially in pulmonary infections, therapeutic regimens include extended complication programs with several reported adverse effects [1,57,58]. However, the therapeutic overlap between antituberculosis treatment and antibiotics for melioidosis poses challenges, as adverse drug reactions and drug-drug interactions may compromise patient adherence and efficacy, which should be further investigated.

*Leptospira* spp. coinfection [5,6,10,26,27,37] had the highest reported mortality rate (75%), which was the highest report for other coinfections. The pathogenic synergy between *Leptospira* and *B. pseudomallei* likely stems from their shared capacity to cause severe systemic inflammation [1,2,59,60], particularly in tropical regions where both pathogens cocirculate. *Leptospira* is known for inducing endothelial damage and vasculitis, which can exacerbate sepsis, renal failure, and disseminated intravascular coagulation [59,60], all of which are common complications of melioidosis [1,2]. The presence of *Leptospira* spp. may also complicate the diagnostic process, as both pathogens can cause overlapping syndromes, including acute febrile illness with multiorgan involvement [60,61]. The high mortality rate associated with *Leptospira* coinfection underscores the need for heightened clinical vigilance and prompt initiation of empirical treatment covering both pathogens, particularly in patients presenting with renal dysfunction or signs of vascular compromise. Preventive measures, such as rodent control and improved sanitation, may reduce the incidence of *Leptospira*-related complications.

Although less frequently reported, viral coinfections, including HIV, have implications for melioidosis outcomes. HIV coinfection [7,21,22,23,30] is associated with a relatively low mortality rate (16.7%), which may reflect improved antiretroviral therapy (ART) coverage in recent years. However, the immunocompromised state induced by HIV, particularly in individuals with low CD4 counts, predisposes patients to disseminated melioidosis and poor outcomes [7]. Other viral pathogens, such as dengue virus, have also been documented in the literature [20]. Dengue virus coinfection may compound endothelial dysfunction and vascular permeability [62], which are central to dengue hemorrhagic fever and septic shock in melioidosis. Coinfections with viral agents require careful hemodynamic monitoring and supportive care to mitigate overlapping pathophysiological effects.

Although less common, fungal and protozoal coinfections pose unique challenges in melioidosis management. Coinfections with fungal species, such as Cryptococcus neoformans, can lead to severe meningoencephalitis in immunosuppressed individuals, such as patients with systemic lupus erythematosus (SLE) [18]. Similarly, protozoal pathogens such as Leishmania may further impair host immune responses [25], potentially accelerating the progression of melioidosis. The mortality associated with these rare coinfections underscores the importance of tailored antifungal or antiparasitic therapy, alongside standard treatment for *B. pseudomallei*. These cases highlight the need for increased clinical awareness and thorough testing in endemic regions.

Despite the significant overlap in risk factors, such as diabetes and agricultural exposure, the mortality rate among tuberculosis coinfected patients (22.2%) is lower than that for other pathogens, potentially reflecting advances in tuberculosis management in endemic regions. Diabetes mellitus, a significant risk factor for melioidosis [3,33,34,38], was not significantly associated with mortality in this study. This finding might be attributed to the inclusion of adjusted models that account for confounding factors. Nonetheless, diabetes remains a critical consideration owing to its impact on the immune system [63], mainly through impaired neutrophil function and delayed clearance of infections [64].

Socioeconomic disparities were evident in this analysis, with higher-income countries contributing more single-infection studies and lower-income countries reporting a greater prevalence of coinfections. This disparity highlights how healthcare access and reporting practices affect outcomes. In resource-limited settings, delays in diagnosis and poor treatment can worsen results for co-infected patients. Furthermore, the observed trend of reduced mortality in lower-middle-income countries might reflect survivor bias, unaccounted confounders, or variability in healthcare staff competencies [65,66]. A previous study by Hanson et al. (2021) reported that melioidosis is a disease of socioeconomic disadvantage and socioeconomic inequality increases disease risk [67].

The associations between agricultural occupation and melioidosis-related mortality were also analyzed. The agricultural occupation exhibited a non-significant association with mortality of melioidosis in this report with OR = 0.82 (95% CI: 0.32–2.07, p = 0.669). However, agricultural workers and farm animals face increased exposure to *B. pseudomallei* in soil and water [1,2,3,68], particularly during monsoon seasons. Preventive measures, such as awareness, personal-protective clothing, and targeted public health campaigns, could mitigate this occupational hazard.

This study’s comprehensive approach to synthesizing data across multiple contexts is a key strength, providing a more complete understanding of prognostic factors in melioidosis. The use of causal diagrams and sensitivity analyses strengthens the findings by addressing biases related to missing data and confounding variables. However, the study has several limitations. First, the reliance on observational studies may introduce selection and reporting biases. While DAGs were used to guide confounding adjustment, causal associations may not be established with high certainty and may require careful consideration. Second, heterogeneity across studies, particularly due to differences in defining coinfection and diagnostic criteria, may affect the generalisability of the findings. We accounted for this variability using multilevel mixed-effects logistic regression models, which improved the robustness of the estimates. However, inconsistencies in study definitions remain a limitation. Third, the small sample size for certain coinfections resulted in wide confidence intervals, limiting the precision of these estimates. Fourth, publication bias was not assessed, as it is not typically applicable to IPD meta-analyses that include case reports or case series. However, sensitivity analysis using multiple imputations suggested that the results remain robust. Finally, the number of individual patients analysed (*n* = 509) appears small compared to the total number of studies included (346). This reflects the limited availability of studies with individual patient data rather than a weakness in the analysis itself.

These findings underscore the urgent need for enhanced diagnostic and therapeutic strategies tailored to high-risk groups, particularly those with disseminated disease and coinfections. The gold standard method, bacteria culturation, is time consummation (taking longer than three days). However, current automated and non-automated methods, which reduce diagnosis time, have reported varying accuracies [69,70]. The incorporation of molecular diagnostics [69] and host-response biomarkers [70] into clinical workflows could improve early detection and risk stratification. Additionally, multidisciplinary strategies that address socioeconomic barriers provide occupational protection, and raise awareness in endemic areas are necessary.

## 5. Conclusions

This study comprehensively analyzed the prognostic factors influencing mortality in patients with melioidosis, focusing on disseminated disease and coinfections. Disseminated melioidosis significantly increases the risk of mortality, highlighting its critical role as the most impactful prognostic factor. The systemic spread of *Burkholderia pseudomallei* emphasizes the importance of early diagnosis and aggressive management strategies to improve survival rates.

Coinfections were observed in 10.8% of patients with Mycobacterium tuberculosis and *Leptospira* spp. being the most common co-pathogens. While tuberculosis coinfection was associated with a modest mortality risk (22.2%), *Leptospira* spp. coinfection had an alarmingly high fatality rate (75%). These findings indicate that specific coinfections may worsen disease severity through combined effects, requiring rapid recognition and intervention.

Demographic and socioeconomic factors, particularly in lower-middle income settings and agricultural occupations, highlight the influence of environmental exposures and healthcare disparities on outcomes. Although diabetes mellitus and other underlying conditions were prevalent, their associations with mortality were not statistically significant, likely due to confounding adjustments in the analysis.

This study highlights the need for practical diagnostic tools, particularly in endemic regions, to identify disseminated cases and coinfections at an early stage. Public health interventions for high-risk populations, improved healthcare access, and further research into melioidosis are crucial for reducing mortality. These findings point to critical areas for clinical and public health improvements to enhance survival outcomes in affected populations.

## Figures and Tables

**Table 1:** Study- and patient-level characteristic and the frequency of reporting study.

Characteristics	Unreported Data *n* (%)	All Patients (*n* = 509)	Co-Infection (*n* = 55)	Single-Infection (*n* = 454)
**Number of studies**		346	32	314
**Study-level characteristics**				
**Year of publication**	0 (0.0)			
1998–2015		212 (41.7)	32 (58.2)	180 (39.6)
2016–2023		297 (58.4)	23 (41.8)	274 (60.4)
**Socio-economic country status**	0 (0.0)			
High income		167 (32.9)	4 (7.2)	163 (35.9)
Upper-middle income		166 (32.7)	36 (35.5)	130 (28.6)
Lower-middle income		175 (34.5)	15 (27.3)	160 (3.5)
**Patient-level characteristics**				
**Age (year)**	0 (0.0)	45.4 ± 18.1	39.7 ± 18.4	46.1 ± 18.1
≤18		60 (11.9)	7 (12.7)	53 (11.7)
19–49		217 (43.0)	26 (47.3)	191 (42.1)
≥50		228 (45.1)	22 (40.0)	206 (45.4)
**Sex**	0 (0.0)			
Male		357 (70.1)	45 (81.8)	312 (68.7)
Female		152 (29.9)	10 (18.2)	142 (31.3)
**Agricultural occupation**	309 (60.7)			
Related		96 (18.9)	19 (34.5)	77 (17.0)
Not related		104 (20.4)	12 (21.8)	92 (2.0)
**Diabetes mellitus**	1 (0.2)			
Presence		262 (51.5)	30 (54.5)	232 (51.1)
Absence		246 (48.3)	24 (43.6)	222 (48.9)
**Other underlying disease** ^a^	1 (0.2)			
Presence		200 (39.3)	16 (29.1)	184 (40.5)
Absence		308 (60.5)	38 (69.1)	270 (5.9)
**Type of infection**	0 (0.0)			
Single-infection		454 (89.2)	0	454 (100)
Co-infection		55 (10.8)	55 (100)	0
**Dissemination of disease ^b^**	3 (0.6)			
Disseminated		286 (56.2)	34 (61.8)	252 (55.5)
Non-disseminated		220 (43.2)	18 (32.7)	202 (44.5)

^a^ systemic lupus erythematosus, alcoholism, malignancy, active smoker, hypertension, obesity, coronary artery disease, congestive cardiac failure, chronic obstructive pulmonary disease (COPD), thyrotoxicosis, ischemic heart disease, hyperlipidemia, gout. ^b^ patient with septicemia caused by *B. pseudomallei* or multiple sites of positive culture.

**Table 2:** Death proportion for each co-infected organism.

Co-Infection Organism	Alive *n* (%)	Death *n* (%)
TB	14 (77.8)	4 (22.2)
*Leptospira*	3 (25.0)	9 (75.0)
HIV	10 (83.3)	2 (16.7)
Other bacteria	3 (75.0)	1 (25.0)
Other viruses	8 (66.7)	4 (33.3)
Other mycobacteria	2 (100.0)	0 (0.0)
Fungus/yeast	1 (50.0)	1 (50.0)
Protozoa/Trematodes	2 (100.0)	0 (0.0)

Abbreviation: TB—tuberculosis; HIV—Human immunodeficiency virus.

**Table 3:** Death proportion of prognosis factors and multivariable analyses with and without imputation, based on conceptual causal diagram.

Prognosis Factor	Alive	Death	Adjusted OR, CCA (95% CI)	*p* Value	Adjusted OR, MI (95% CI)	*p* Value
**Model 1** **Publication year**			(*n* = 509, 100%)			
1998–2015	160 (75.5)	52 (24.5)	Reference		NA	
2016–2023	233 (78.5)	64 (21.5)	0.88 (0.36–2.16)	0.781	NA	NA
**Model 2** **Socio-economic country status**			(*n* = 508, 99.9%)		(*n* = 509, 100.0%)	
High income	131 (78.4)	36 (21.6)	Reference		Reference	
High-middle income	118 (71.1)	48 (28.9)	2.11 (0.73–6.05)	0.166	2.11 (0.73–6.05)	0.166
Low-middle income	143 (81.7)	32 (18.3)	1.03 (0.36–3.00)	0.950	1.03 (0.36–3.00)	0.950
**Model 3** **Age groups**			(*n* = 505, 98.8%)		(*n* = 509, 100.0%)	
Age ≤ 19	45 (75.0)	15 (25.0)	Reference		Reference	
Age 20–49	168 (77.4)	49 (22.6)	0.62 (0.17–2.34)	0.483	0.62 (0.17–2.37)	0.489
Age ≥ 50	177 (77.6)	51 (21.4)	0.50 (0.13–1.94)	0.314	0.50 (0.13–1.98)	0.327
**Model 4** **Sex**			(*n* = 509, 100.0%)			
Female	82 (76.6)	25 (23.4)	Reference		NA	
Male	274 (79.0)	73 (21.0)	0.67 (0.30–1.48)	0.319	NA	NA
**Model 5** **Agricultural occupation**			(*n* = 199, 39.0%)		(*n* = 509, 100.0%)	
Not related	82 (78.9)	22 (21.2)	Reference		Reference	
Related	76 (79.2)	20 (20.8)	0.82 (0.32–2.07)	0.669	0.78 (0.29–2.16)	0.632
**Model 6** **Diabetes mellitus**			(*n* = 503, 98.8%)		(*n* = 509, 100.0%)	
Absence	194 (78.9)	52 (21.1)	Reference		Reference	
presence	199 (76.0)	63 (24.0)	1.35 (0.60–3.05)	0.471	1.36 (0.60–3.07)	0.461
**Model 7** **Other underlying**			(*n* = 508, 99.9%)		(*n* = 509, 100.0%)	
Absence	247 (80.2)	61 (19.8)	Reference		Reference	
presence	146 (73.0)	54 (27.0)	1.57 (0.76–3.24)	0.225	1.59 (0.77–3.27)	0.212
**Model 8** **Type of infection**			(*n* = 503, 98.8%)		(*n* = 509, 100.0%)	
Single-infection	356 (78.4)	98 (21.6)	Reference		Reference	
Co-infection	37 (67.3)	18 (32.7)	2.70 (0.53–13.90)	0.235	3.17 (0.60–16.62)	0.172
**Model 9** **Dissemination of disease**			(*n* = 500, 98.2%)		(*n* = 509,100.0%)	
Non disseminated	191 (86.8)	29 (13.2)	Reference		Reference	
Disseminated	200 (69.9)	86 (30.1)	4.93 (2.14–11.37)	<0.001	4.70 (2.06–10.69)	<0.001

Abbreviation: CCA, complete case analysis; CI, confident interval, MI, multiple imputation. NA: Not Applicable.

## Data Availability

No new data were created or analyzed in this study. Data sharing is not applicable to this article.

## Supplementary Materials

The following supporting information can be downloaded at: https://www.mdpi.com/article/10.3390/epidemiologia6020017/s1, Table S1: Causal modeling of each prognosis factor and the validity of the complete case analysis; Table S2: The strength of supporting evidence in the conceptual causal diagram; Table S3: Sensitivity analysis based on only the evidential support causal diagram; Figures S1–S10: Causal diagram of Model 1−10; Supplementary File S1: References list for 651 single-infected patients; Supplementary File S2: Details of the data extracted from the coinfection and single-infection. Cited references [71,72,73,74,75,76,77,78,79,80,81,82,83,84,85].
