# Supplementary material for: Co-Infections and Their Prognostic Impact on Melioidosis Mortality: A Systematic Review and Individual Patient Data Meta-Analysis
Source: Epidemiologia (Basel). 2025 Apr 1;6(2):17. doi: 10.3390/epidemiologia6020017 (PMC12015870; doi:10.3390/epidemiologia6020017)
Supplement: Supplementary file 1 [file epidemiologia-06-00017-s001.zip › epidemiologia-3508647-supplementary conversion.pdf]

epidemiologia

MDPI

Supplementary Materials

Table S1. Causal modeling of each prognosis factor and the validity of the complete case analysis.

Model number	Factor	Causal Adjusted set	CCA analysis	Factor associated with missing data
1	Year of publication	None	Valid	None
2	Socioeconomic country status	None	Valid	None
3	Age	None	Valid	None
4	Sex	None	Valid	None
5	Agricultural Occupation	Age, Socioeconomic country status	Valid	Age, Coinfection, Dissemination of disease, Socioeconomic country status
6	Diabetes mellitus	Age, Sex, Socioeconomic country status	Valid	None
7	Other underlying disease	None	Valid	None
8	Type of infection	Age, DM, Sex, Socioeconomic country status	Valid	None
9	Dissemination of disease	Age, DM, Sex, Socioeconomic country status	Valid	None

Abbreviation: CCA, complete case analysis; DM, diabetes mellitus.

Table S2. The strength of supporting evidence in the conceptual causal diagram.

Relations between variable	Supporting evidence	Consider sensitivity analysis
Year of publication causally impacts on death rate in patient with melioidosis	Indirect with biological plausibility [1, 2]	Research objective
Socioeconomic country status causally impacts on death rate in patient with melioidosis	Indirect with biological plausibility [3, 4]	Research objective
Socioeconomic country status causally impacts on proportion of patient with diabetes	Direct evidence [5]	No
Socioeconomic country status causally impacts on proportion of Agricultural occupation	Indirect with biological plausibility [6, 7]<br>Data availability	No
Socioeconomic country status causally impacts on missingness of Agricultural occupation data	Evidence from available data	Yes, by multiple imputation
Age groups causally impact on death rate in patient with melioidosis	Weak direct evidence [8–10]	Research objective
Age groups causally impact on proportion of Agricultural occupation	expert opinions	Yes, by removing the relationship
Age groups causally impact on missingness of Agricultural occupation data	Evidence from available data	Yes, by multiple imputation

Age groups causally impact on proportion of patient with diabetes	Well known fact	No
Age groups causally impact on proportion of patient with dissemination disease	Indirect with biological plausibility [11]	No
Sex causally impacts death rate in patient with melioidosis	Indirect with biological plausibility [12]	Research objective
Sex causally impacts on proportion of patient with diabetes	Strong direct evidence [13–15]	No
Sex causally impacts on proportion of patient with coinfection type	Evidence from available data, expert opinion	Yes, by removing the relationship
Agricultural occupation causally impacts on proportion of patient with coinfection type	expert opinion	Yes, by removing the relationship
Diabetes causally impacts on death rate in patient with melioidosis	Strong direct evidence [8–10]	Research objective
Diabetes causally impacts on proportion of patient with coinfection type	Indirect with biological plausibility [16]	No
Diabetes causally impacts on proportion of patient with dissemination disease	Indirect with biological plausibility [16]	No
Other underlying causally impacts on death rate in patient with melioidosis	direct evidence	Research objective
Coinfection type causally impacts on death rate in patient with melioidosis	Indirect with biological plausibility [17]	Research objective
Coinfection type causally impacts on missingness of Agricultural occupation data	Evidence from available data	Yes, by multiple imputation
Dissemination of disease causally impacts on death rate in patient with melioidosis	Strong direct evidence [18]	Research objective
Dissemination of disease causally impacts on missingness of Agricultural occupation data	Evidence from available data	Yes, by multiple imputation

Table S3. Sensitivity analysis based on only the evidential support causal diagram.

Prognosis factor	Causal Adjusted set	Adjusted OR, CCA (95% CI)	P value	Adjusted OR, MI (95% CI)	P value
**Model 1**					
**Publication year**		(*n* = 509, 100%)			
1998-2015	No change	Reference		NA	
2016-2023		No change		NA	NA
**Model 2**					
**Socioeconomic country status**		(*n* = 508, 99.9%)		(*n* = 509, 100.0%)	
High income		Reference		Reference	
High-middle income	No change	No change		No change	
Low-middle income		No change		No change	
**Model 3**					
**Age groups**		(*n* = 505, 98.8%)		(*n* = 509, 100.0%)	
Age ≤19		Reference		Reference	
Age 20-49	No change	No change		No change	
Age ≥50		No change		No change	

**Model 4**					
**Sex**		(n = 509, 100.0%)			
Female	No change	Reference		NA	
Male		No change		NA	NA
**Model 5**					
**Agricultural occupation**		(n = 199, 39.0%)		(n = 509, 100.0%)	
Not related	Socioeconomic country status	Reference		Reference	
Related		0.79 (0.32-1.95)	0.615	0.76 (0.29-1.98)	0.564
**Model 6**					
**Diabetes mellitus**		(n = 503, 98.8%)		(n = 509, 100.0%)	
Absence	No change	Reference		Reference	
presence		No change		No change	
**Model 7**					
**Other underlying**		(n = 508, 99.9%)		(n = 509, 100.0%)	
Absence	No change	Reference		Reference	
presence		No change		No change	
**Model 8**					
**Type of infection**		(n = 503, 98.8%)		(n = 509, 100.0%)	
Single-infection	No change	Reference		Reference	
Coinfection		No change		No change	
**Model 9**					
**Dissemination of disease**		(n = 500, 98.2%)		(n = 509,100.0%)	
Non disseminated	No change	Reference		Reference	
Disseminated		No change		No change	

Abbreviation: CCA, complete case analysis; CI, confident interval, MI, multiple imputation.

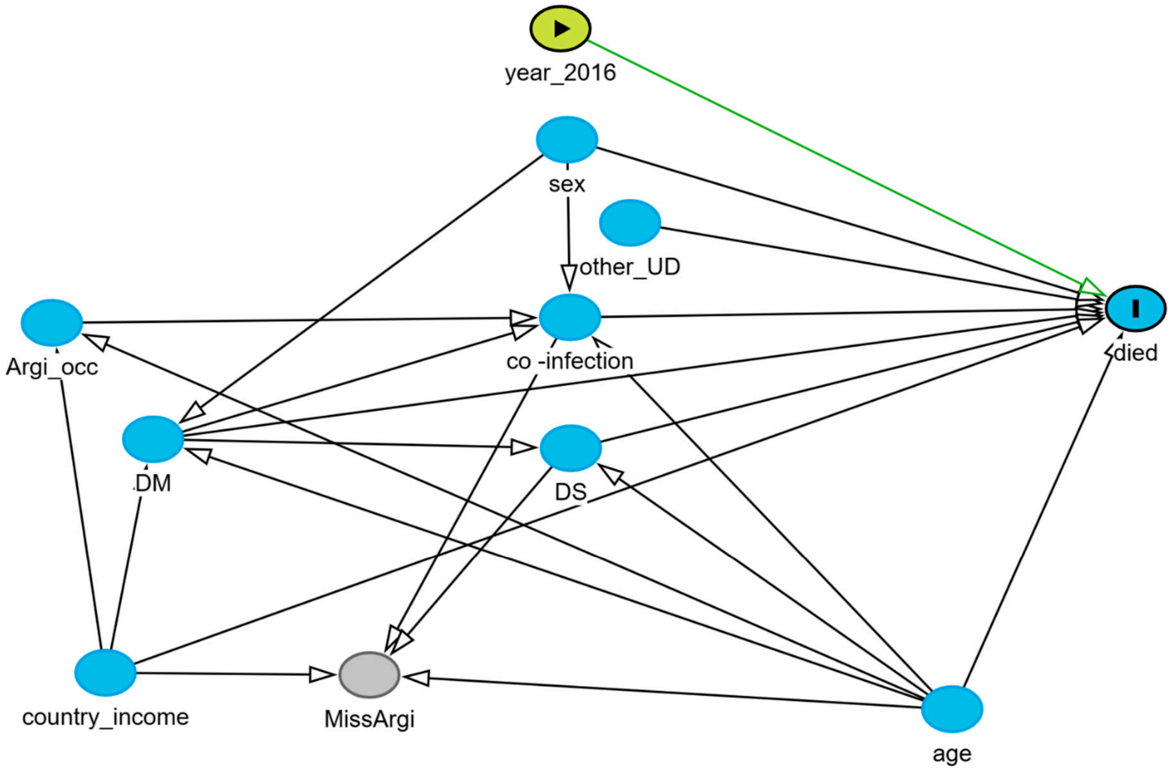

Figure S1. Causal diagram of Model 1. A diagram showing a causal relationship between observed year_2016, a binary variable that indicates whether studies are published in older or later 2015 and observed outcome (died) of multilevel logistic regression. Green arrow indicates causal pathway. MissArgi, a binary variable that indicates whether agricultural occupation is observed or missing. Abbreviation: Argi_occ, agricutural occupation; country_income, socioeconomics country status;DS, disseminated, DM, diabetes mellitus, other_UD, other underlying disease; year_2016, the publication year after 2015

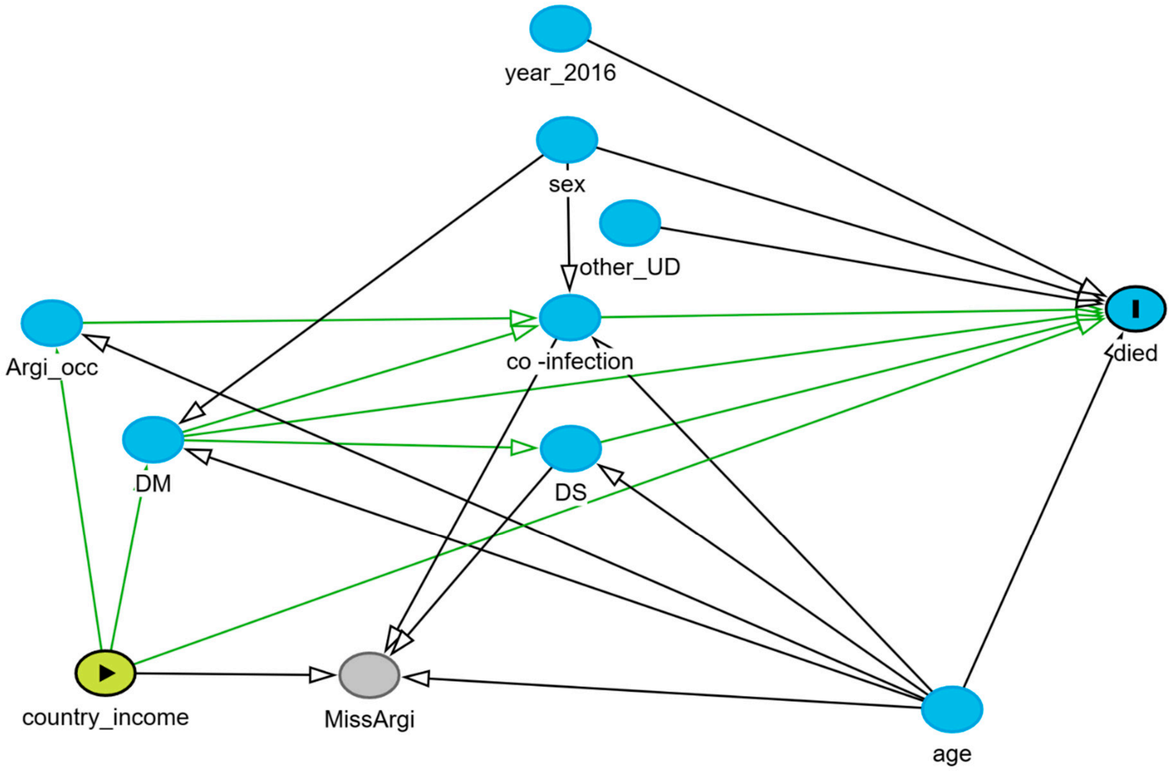

Figure S2. Causal diagram of Model 2. A diagram showing a causal relationship between observed Socioeconomic country status and observed outcome (died) of multilevel logistic regression. Green arrow indicates causal pathway. MissArgi, a binary variable that indicates whether agricultural occupation is observed or missing. Abbreviation: Argi_occ, agricutural occupation; country_income, socioeconomics country status;DS, disseminated, DM, diabetes mellitus, other_UD, other underlying disease; year_2016, the publication year after 2015

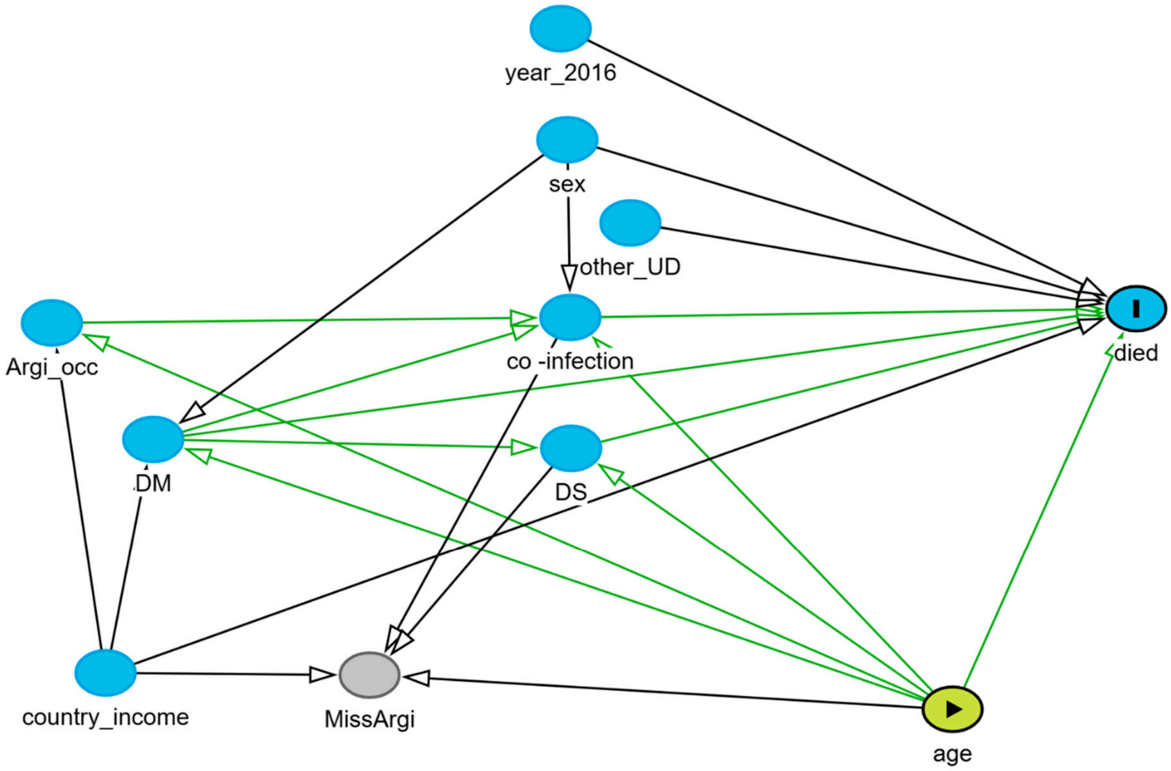

Figure S3. Causal diagram of Model 3. A diagram showing a causal relationship between observed age groups and observed outcome (died) of multilevel logistic regression. Green arrow indicates causal pathway. MissArgi, a binary variable that indicates whether agricultural occupation is observed or missing. Abbreviation: Argi_occ, agricutural occupation; country_income, socioeconomics country status;DS, disseminated, DM, diabetes mellitus, other_UD, other underlying disease; year_2016, the publication year after 2015

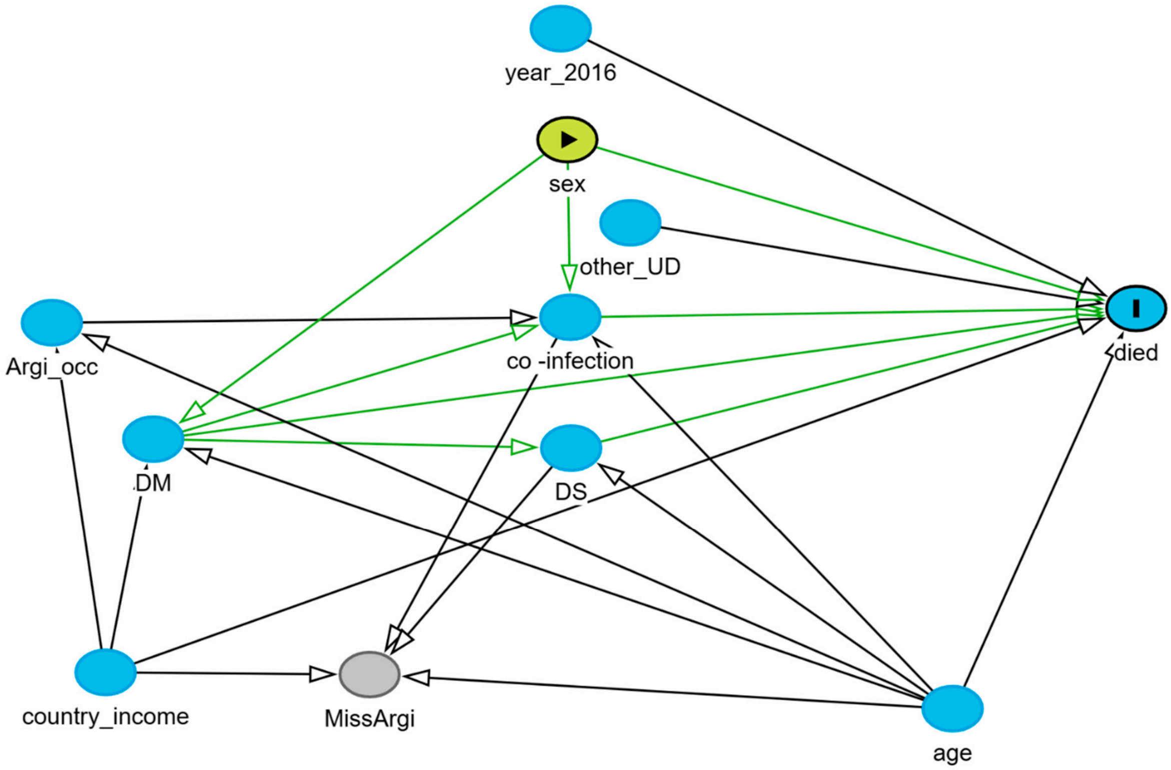

Figure S4. Causal diagram of Model 4. A diagram showing a causal relationship between observed sex and observed outcome (died) of multilevel logistic regression. Green arrow indicates causal pathway. MissArgi, a binary variable that indicates whether agricultural occupation is observed or missing. Abbreviation: Argi_occ, agricutural occupation; country_income, socioeconomics country status;DS, disseminated, DM, diabetes mellitus, other_UD, other underlying disease; year_2016, the publication year after 2015

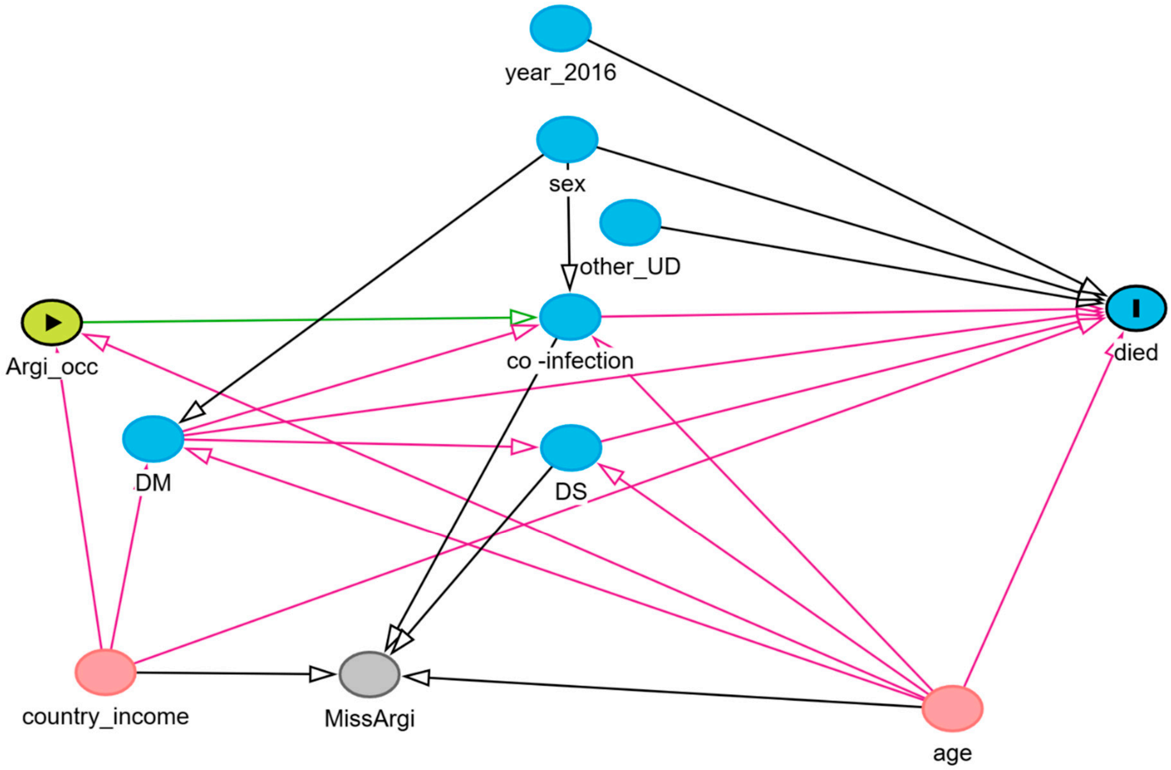

Figure S5. Causal diagram of Model 5. A diagram showing a causal relationship between observed agricultural occupation and observed outcome (died) of multilevel logistic regression. Green arrow indicates causal pathway and pink arrow indicates bias pathway. MissArgi, a binary variable that indicates whether agricultural occupation is observed or missing. Abbreviation: Argi_occ, agricutural occupation; country_income, socioeconomics country status;DS, disseminated, DM, diabetes mellitus, other_UD, other underlying disease; year_2016, the publication year after 2015

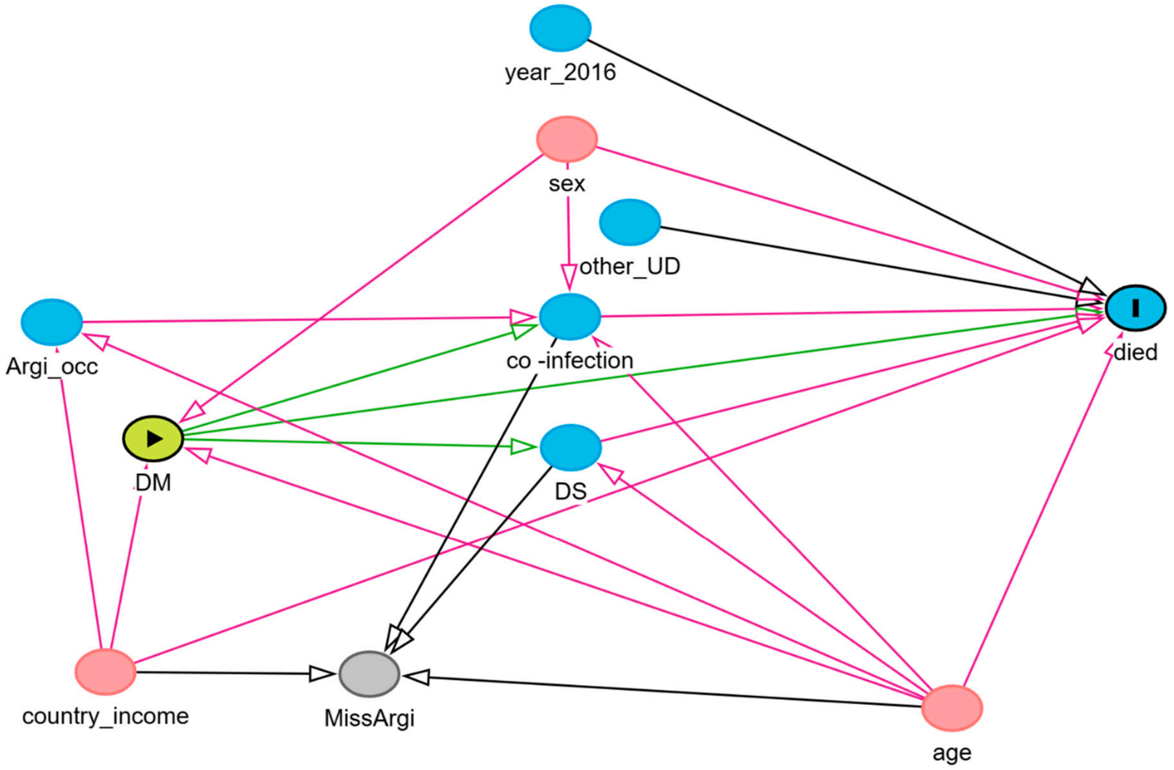

Figure S6. Causal diagram of Model 6. A diagram showing a causal relationship between observed diabetes mellitus and observed outcome (died) of multilevel logistic regression. Green arrow indicates causal pathway and pink arrow indicates bias pathway. MissArgi, a binary variable that indicates whether agricultural occupation is observed or missing. Abbreviation: Argi_occ, agricutural occupation; country_income, socioeconomics country status;DS, disseminated, DM, diabetes mellitus, other_UD, other underlying disease; year_2016, the publication year after 2016

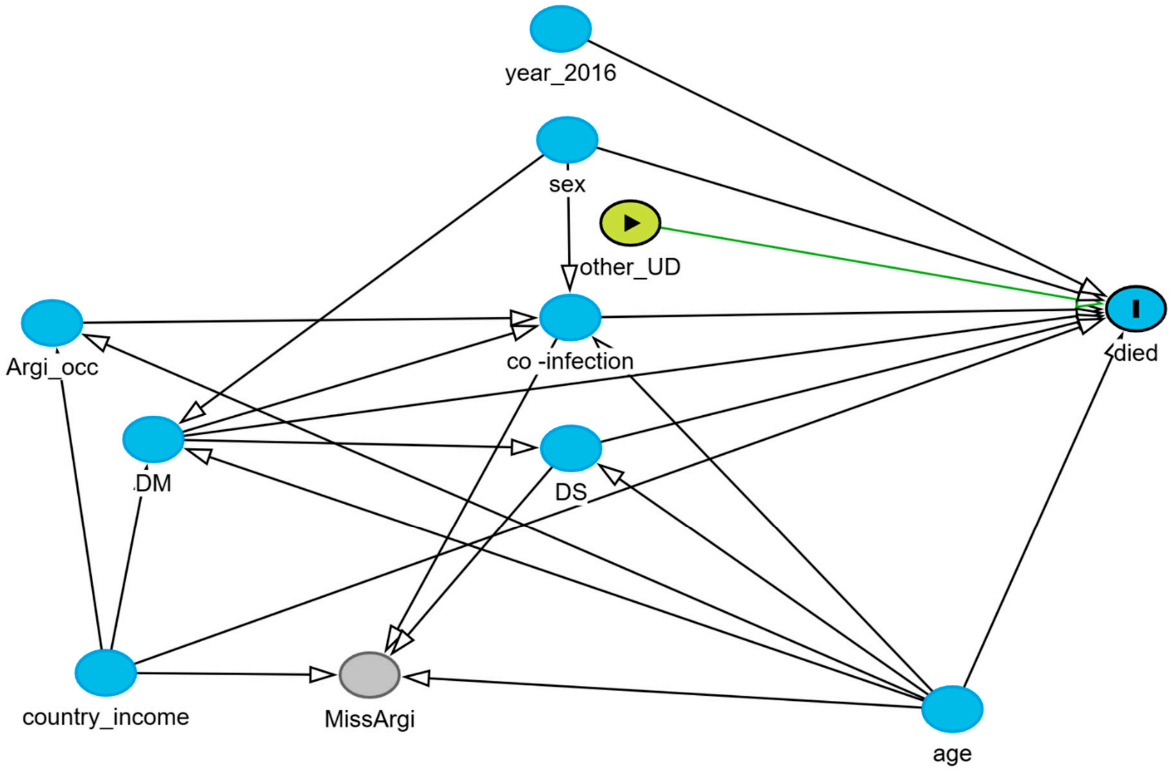

Figure S7. Causal diagram of Model 7. A diagram showing a causal relationship between observed other underlying diseases and observed outcome (died) of multilevel logistic regression. Green arrow indicates causal pathway. MissArgi, a binary variable that indicates whether agricultural occupation is observed or missing. Abbreviation: Argi_occ, agricutural occupation; country_income, socioeconomics country status;DS, disseminated, DM, diabetes mellitus, other_UD, other underlying disease; year_2016, the publication year after 2015

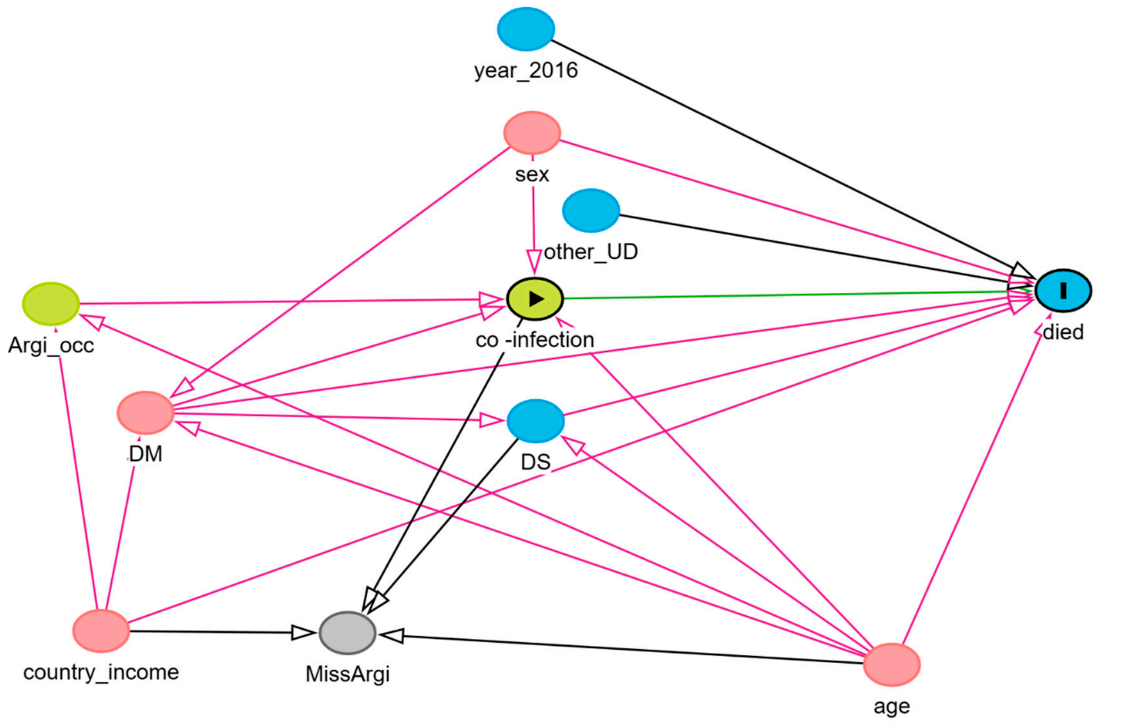

Figure S8. Causal diagram of Model 8. A diagram showing a causal relationship between observed status of coinfection and observed outcome (died) of multilevel logistic regression. Green arrow indicates causal pathway and pink arrow indicates bias pathway. MissArgi, a binary variable that indicates whether agricultural occupation is observed or missing. Abbreviation: Argi_occ, agricutural occupation; country_income, socioeconomics country status;DS, disseminated, DM, diabetes mellitus, other_UD, other underlying disease; year_2016, the publication year after 2015

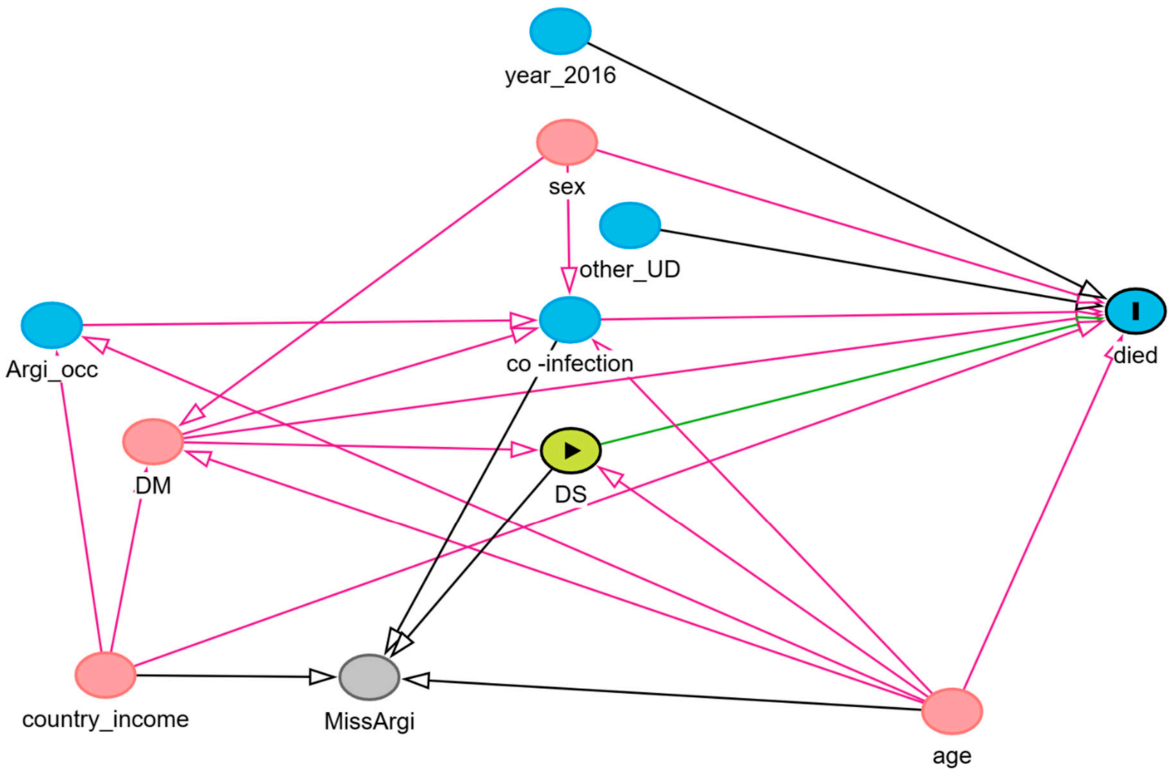

Figure S9. Causal diagram of Model 9. A diagram showing a causal relationship between observed dissemination of disease and observed outcome (died) of multilevel logistic regression. Green arrow indicates causal pathway and pink arrow indicates bias pathway. MissArgi, a binary variable that indicates whether agricultural occupation is observed or missing. Abbreviation: Argi_occ, agricutural occupation; country_income, socioeconomics country status;DS, disseminated, DM, diabetes mellitus, other_UD, other underlying disease; year_2016, the publication year after 2015

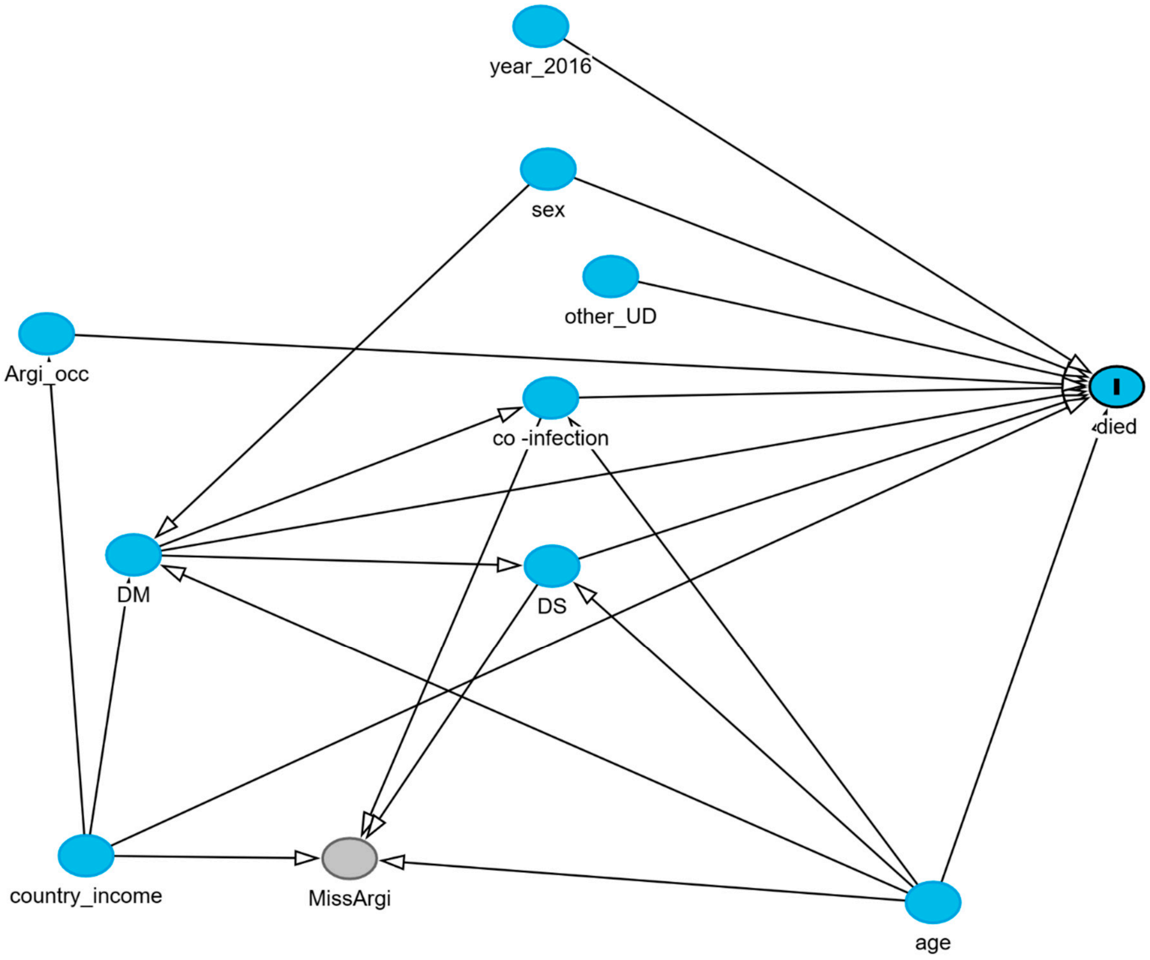

Figure S10. Only Evidential support causal diagram. A diagram showing a causal relationship between each two variables indicated by black arrow MissArgi, a binary variable that indicates whether agricultural occupation is observed or missing. Abbreviation: Argi_occ, agricutural occupation; country_income, socioeconomics country status;DS, disseminated, DM, diabetes mellitus, other_UD, other underlying disease; year_2016, the publication year after 2015

References

71. Pitman, M.C.; Luck, T.; Marshall, C.S.; Anstey, N.M.; Ward, L.; Currie, B.J. Intravenous therapy duration and outcomes in melioidosis: A new treatment paradigm. *PLoS Neglected Trop. Dis.* **2015**, *9*, e0003586. https://doi.org/10.1371/journal.pntd.0003586 PMID: 25811783.
72. Sullivan, R.P.; Marshall, C.S.; Anstey, N.M.; Ward, L.; Currie, B.J. 2020 Review and revision of the 2015 Darwin melioidosis treatment guideline; paradigm drift not shift. *PLOS Neglected Trop. Dis.* **2020**, *14*, e0008659. https://doi.org/10.1371/journal.pntd.0008659.
73. Marmot, M. The Health Gap: The Challenge of an Unequal World: The argument. *Int J Epidemiol.* **2017**, *46*, 1312–1318. https://doi.org/10.1093/ije/dyx163. PMID: 28938756; PMCID: PMC5837404.

74. Richards, S.E.; Wijeweera, C.; Wijeweera, A. Lifestyle and socioeconomic determinants of diabetes: Evidence from country-level data. *PLoS ONE.* **2022**, *17*, e0270476. https://doi.org/10.1371/journal.pone.0270476. PMID: 35901054; PMCID: PMC9333224.
75. Sharma, S.; Yadav, P.K.; Dahal, R.; Shrestha, S.K.; Bhandari, S.; Thapaliya, K.P. Agriculture in relation to socioeconomic status of Tharu in Chitwan of Nepal. *J. Agric. Food Res.* **2021**, *6*, 100243.
76. Fujishiro, K.; Xu, J.; Gong, F. What does "occupation" represent as an indicator of socioeconomic status?: Exploring occupational prestige and health. *Soc. Sci. Med.* **2010**, *71*, 2100–2107.
77. Hassan, M.R.; Pani, S.P.; Peng, N.P.; Voralu, K.; Vijayalakshmi, N.; Mehanderkar, R.; Aziz, N.A.; Michael, E. Incidence, risk factors and clinical epidemiology of melioidosis: A complex socio-ecological emerging infectious disease in the Alor Setar region of Kedah, Malaysia. *BMC Infect Dis.* **2010**, *10*, 302. https://doi.org/10.1186/1471-2334-10-302. PMID: 20964837; PMCID: PMC2975659.
78. Mardhiah, K.; Wan-Arfah, N.; Naing, N.N.; Hassan, M.R.A.; Chan, H.K. The Cox model of predicting mortality among melioidosis patients in Northern Malaysia: A retrospective study. *Medicine* **2021**, *100*, e26160. https://doi.org/10.1097/MD.0000000000026160. PMID: 34160382; PMCID: PMC8238369.
79. Menon, R.; Baby, P.; Kumar, V.A.; Surendran, S.; Pradeep, M.; Rajendran, A.; Suju, G.; Ashok, A. Risk Factors for Mortality in Melioidosis: A Single-Centre, 10-Year Retrospective Cohort Study. *ScientificWorldJournal* **2021**, *2021*, 8154810. https://doi.org/10.1155/2021/8154810. PMID: 34285680; PMCID: PMC8275413.
80. Michels, E.H.; Butler, J.M.; Reijnders, T.D.; Cremer, O.L.; Scicluna, B.P.; Uhel, F.; Peters-Sengers, H.; Schultz, M.J.; Knight, J.C.; van Vught, L.A; et al. Association between age and the host response in critically ill patients with sepsis. *Crit. Care* **2022**, *26*, 385. https://doi.org/10.1186/s13054-022-04266-9.
81. Dias, S.P.; Brouwer, M.C.; van de Beek, D. Sex and Gender Differences in Bacterial Infections. *Infect Immun.* **2022**, *90*, e0028322. https://doi.org/10.1128/iai.00283-22. PMID: 36121220; PMCID: PMC9584217.
82. Ding, E.L.; Song, Y.; Malik, V.S.; Liu, S. Sex differences of endogenous sex hormones and risk of type 2 diabetes: A systematic review and meta-analysis. *JAMA.* **2006**, *295*, 1288–1299. https://doi.org/10.1001/jama.295.11.1288. PMID: 16537739.
83. Alrashed, F.A.; Iqbal, M.; Alsubiheen, A.M.; Ahmad, T. Exploring determinants of sex and family history-based disparity in type 2 diabetes mellitus prevalence among clinical patients. *BMC Public Health* **2024**, *24*, 682. https://doi.org/10.1186/s12889-024-18170-0.
84. Kronsteiner, B.; Chaichana, P.; Sumonwiriya, M.; Jenjaroen, K.; Chowdhury, F.R.; Chumseng, S.; Teparrukkul, P.; Limmathurotsakul, D.; Day, N.P.J.; Klenerman, P.; Dunachie, S.J. Diabetes alters immune response patterns to acute melioidosis in humans. *Eur J Immunol.* **2019**, *49*, 1092–1106. https://doi.org/10.1002/eji.201848037. PMID: 31032897; PMCID: PMC6618312.
85. Raj, S.; Sistla, S.; Sadanandan, D.M.; Kadhiravan, T.; Rameesh, B.M.S.; Amalnath, D. Clinical Profile and Predictors of Mortality among Patients with Melioidosis. *J Glob Infect Dis.* **2023**, *15*, 72–78. https://doi.org/10.4103/jgid.jgid_134_22. PMID: 37469465; PMCID: PMC10353644.

Disclaimer/Publisher's Note: The statements, opinions and data contained in all publications are solely those of the individual author(s) and contributor(s) and not of MDPI and/or the editor(s). MDPI and/or the editor(s) disclaim responsibility for any injury to people or property resulting from any ideas, methods, instructions or products referred to in the content.
